# The Prevalence of Islet Autoantibodies in Children and Adolescents With Type 1 Diabetes Mellitus: A Global Scoping Review

**DOI:** 10.3389/fendo.2022.815703

**Published:** 2022-02-03

**Authors:** Carlo Ross, Zachary J. Ward, Apoorva Gomber, Maira Owais, Jennifer M. Yeh, Ché-L. Reddy, Rifat Atun

**Affiliations:** ^1^ Department of Global Health and Population, Harvard T.H. Chan School of Public Health, Boston, MA, United States; ^2^ Academic Foundation Programme, Manchester University NHS Foundation Trust, Manchester, United Kingdom; ^3^ Centre for Health Decision Science, Harvard T.H. Chan School of Public Health, Boston, MA, United States; ^4^ Department of Biology and Department of Economics, Amherst College, Amherst, MA, United States; ^5^ Division of General Pediatrics, Boston Children’s Hospital and Harvard Medical School, Boston, MA, United States

**Keywords:** type 1 diabetes, children and adolescent, islet autoantibodies, global health, global, scoping review

## Abstract

**Background and Purpose:**

Pancreatic islet autoantibodies (iAb) are the hallmark of autoimmunity in type 1 diabetes. A more comprehensive understanding of the global iAb prevalence could help reduce avertible morbidity and mortality among children and adolescents and contribute to the understanding in the observed differences in the incidence, prevalence and health outcomes of children and adolescents with type 1 diabetes across and within countries. We present the first scoping review that provides a global synthesis of the prevalence of iAb in children and adolescents with type 1 diabetes.

**Research Design and Methods:**

We searched Ovid MEDLINE^®^ with Daily Update, Embase (Elsevier, embase.com) and PubMed (National Library of Medicine -NCBI), for studies pertaining to prevalence in children and adolescents (0-19) with type 1 diabetes published between 1 Jan 1990 and 18 June 2021. Results were synthesized using Covidence systematic review software and meta-analysis was completed using R v3·6·1. Two reviewers independently screened abstracts with a third reviewer resolving conflicts (k= 0·92).

**Results:**

The review revealed 125 studies from 48 different countries, with 92 from high-income countries. Globally, in new-onset type 1 diabetes, IA-2A was the most prevalent iAb 0·714 [95% CI (0·71, 0·72)], followed by ICA 0·681 [95% CI (0·67, 0·69)], ZnT8A was 0·654 [95% CI (0·64, 0·66)], GADA 0·636 [95% CI (0·63, 0·66)] and then IAA 0·424 [95% CI (0·42, 0·43)], with substantial variation across world regions. The weighted mean prevalence of IA-2A was more variable, highest in Europe at 0·749 [95% CI (0·74, 0·76)] followed by Northern America 0·662 [95% CI (0·64, 0·69)], Latin America and the Caribbean 0·632 [95% CI (0·54, 0·72)], Oceania 0·603 [95% CI (0·54, 0·67)], Asia 0·466 [95% CI (0·44, 0·50)] and Africa 0·311 [95% CI (0·23, 0·40)]. In established cases of type 1 diabetes, GADA was the most prevalent iAb 0·407 [95% CI (0·39, 0·42)] followed by ZnT8A 0·322 [95% CI (0·29, 0·36)], IA-2A 0·302 [95% CI (0·29, 0·32)], IAA 0·258 [95% CI (0·24, 0·26)] and ICA 0·145 [95% CI (0·13, 0·16)], again with substantial variation across world regions.

**Conclusion:**

Understanding the global prevalence of iAb in children and adolescents with type 1 diabetes could help with earlier identification of those at-risk of developing type 1 diabetes and inform clinical practice, health policies, resource allocation, and targeted healthcare interventions to better screen, diagnose and manage children and adolescents with type 1 diabetes

## Introduction

Type 1 diabetes is a chronic autoimmune condition characterised by raised blood sugar levels due to insulin deficiency secondary to loss of the pancreatic islet β-cells (1–3). Type 1 diabetes is classified as either autoimmune (autoantibody positive [iAb+]) or idiopathic (autoantibody negative [iAb-]). The primary form of type 1 diabetes is iAb+, where islet autoantibodies (iAb) destroy pancreatic cellular infiltrate (3).

Globally, the incidence of type 1 diabetes is increasing, particularly among children and adolescents living in low-income and middle-income countries (LMICs) (4–6). In continental subgroups (Asia, Africa, Europe and America) the annual incidence of type 1 diabetes is estimated to be around 15 per 100,000, 8 per 100,000, 15 per 100,000 and 20 per 100,000, respectively (7).

Diabetic Ketoacidosis (DKA), a complication of undiagnosed, untreated or poorly managed type 1 diabetes, is experienced by more than 20-80% of children at the onset of disease and can be fatal if not recognised or managed appropriately (8, 9). Even following diagnosis, management challenges remain and are well documented in adolescents, particularly those diagnosed at an early age, citing reasons such as increased social awareness and concern, inclination towards age-related risk taking and a lack of understanding of future health impacts (10).

First described in 1974, iAb are the hallmark of pancreatic autoimmunity in type 1 diabetes and are recognised to precede the clinical onset of the disease (11, 12). The presence of pancreatic iAb characterises the pre-clinical phase of type 1 iAb+ diabetes in child and adolescents (10, 13). Estimates by Bingley et al. suggest that more than 90% of children who develop type 1 diabetes prior to puberty have detectable iAb by five years of age (14). iAb can be used in the staging and stratification of children and adolescents with type 1 diabetes (15, 16) and also provide the opportunity for counselling and possible reduced morbidity by identifying children and adolescents that are at-risk of developing type 1 diabetes or DKA (13, 15, 16). Type 1 diabetes iAb recognise five types: Glutamic Acid Decarboxylase (GADA), Islet Cell Cytoplasmic (ICA), Insulinoma-Associated-2/Tyrosine Phosphatase (IA-2A), Insulin (IAA) and, Zinc Transporter-8 Autoantibodies (ZnT8A) (17).

An important question in the aetiology of type 1 diabetes relates to the role of iAb in the pathogenesis, natural history and care cascade of type 1 diabetes in children and adolescents. Children and adolescents at increased risk of development of type 1 diabetes progress at different rates in immune activation, development of iAb and subsequent stages of type 1 diabetes. The development of two or more iAb is associated with increased blood glucose and ultimately overt symptomatic type 1 diabetes (18). Evidence from prospective cohort studies which screened children with genetic susceptibility to type 1 diabetes suggest that, in general, it is the number of iAb rather than the specific iAb profile that is most predictive of development of type 1 diabetes, with the majority of children and adolescents with two or more iAb developing overt type 1 diabetes (17–19).

While progression to overt type 1 diabetes is faster in children and adolescents with the presence of multiple iAb before three years of age, iAb may develop later in childhood and throughout the adolescent phases. As such, it has been proposed that those at increased risk of developing type 1 diabetes should be tested annually for the development of iAb until they become adults (18).

Since the complications of undiagnosed type 1 diabetes are life-threatening, it is prudent to effectively screen and diagnose children and adolescents with type 1 diabetes before the onset of DKA, though this has proven challenging. Children and adolescents with a first degree relative with type 1 diabetes have a 15-fold increased relative risk of type 1 diabetes, which helps to inform screening policies, but there is scant guidelines on who else to screen and how to identify the population at risk (20–22). Further, although iAb are routinely used to define pre-clinical type 1 diabetes, the reported prevalence of idiopathic type 1 diabetes (iAb-) is high in some studies (23, 24). A global picture of iAb prevalence and profiles with respect to DKA risk, C-peptide levels and associated morbidity and mortality, is lacking from the literature. Determining the patterns and trends of incidence of iAb in type 1 diabetes could improve current understanding of the global population of children and adolescents at-risk of developing type 1 diabetes and contribute to the understanding in the observed differences in the incidence, prevalence and health outcomes of children and adolescents with type 1 diabetes across and within countries. A more comprehensive understanding of the global iAb prevalence could improve the detection and management of type 1 diabetes and provide a foundation for further study into the associated severity of respective autoantibody profiles, helping reduce avertible morbidity and mortality among children and adolescents (25).

The aim of this global scoping review was to determine: 1) the proportion of children and adolescents with type 1 diabetes that have Glutamic Acid Decarboxylase Autoantibodies (GADA), Islet Cell Cytoplasmic Autoantibodies (ICA), Insulinoma-Associated-2 Autoantibodies/Tyrosine Phosphatase (IA-2A), Insulin Autoantibodies (IAA) or Zinc Transporter-8 Autoantibodies (ZnT8A). 2) the proportion of children and adolescents with type 1 diabetes that have no iAb, a single iAb and multiple iAb (among iAb tested); and 3) the variation in the presence of iAb globally in terms of region and income classification for new onset or established type 1 diabetes status.

## Methods

### Overview

A systematic search was conducted to identify literature pertaining to iAb in children and adolescents with type 1 diabetes. Our scoping review was done in accordance with the scoping review guidelines as recommended by Arksey and O’Malley and the Joanna Briggs Institute (26). Data were extracted globally across regional, national and international studies from acute care, community and research based settings (e.g. public health screening of a participant group).

### Data Sources and Searches

Our search was conducted on Ovid MEDLINE^®^ with Daily Update, Embase and PubMed on 19 June 2021, for entries from 1 Jan 1990 to 18 June 2021 using search terms (**Supplementary Material 1**) which were developed with guidance from a librarian from the Harvard T.H. Chan School of Public Health Countway Library. Reference lists of review articles were studied to identify additional literature not identified in the initial database search. Publications from any country were included. However, the language criterion was limited to English.

Publications were deemed eligible for inclusion if the study reported iAb results for children and adolescents (aged 0-19 years) diagnosed with type 1 diabetes. Secondary data collection was performed through a review of existing primary research studies, systematic reviews and meta-analyses. Only quantitative studies were included. Studies were excluded if they focused on type 1 diabetes in children and adolescents but not on iAb. Similarly, studies were excluded if they explored iAb in type 1 diabetes but did not focus on children and adolescents (i.e. an adult population). Articles published before 1990 as well as those that did not study iAb or had no abstract available *via* Covidence or Google were also excluded. Articles that focussed on the relationship between iAb and other autoimmune conditions such as coeliac or thyroid disease were excluded.

### Study Selection

Search results were imported into Covidence reference management software and duplicates were removed. Abstracts were independently screened by two authors (CR and AG) to identify articles meeting inclusion criteria. Disagreements were resolved by consensus with the third author (MO). Full texts were then reviewed by two authors, with final inclusion in the study determined by author consensus. The inter-rater reliability using a 20% randomly selected sample was determined using Cohen’s Kappa co-efficient (k= 0·92).

### Data Synthesis and Analysis

Data were collected using Microsoft Excel 16.51 and statistical analysis was performed using R v3·6·1. iAb studies were subdivided into two groups: ‘new-onset’ type 1 diabetes (iAb tested at diagnosis-12 months following diagnosis) and ‘established’ type 1 diabetes cases (type 1 diabetes duration >12 months) for analysis. We calculated the proportion of patients in each group testing positive for each iAb, and estimated means and 95% uncertainty intervals (calculated from Beta distributions) by geographic area. We also estimated the proportion of patients testing positive for any iAb by geographic area, conditional on the number of iAbs tested for.

## Results

**Figure 1:**
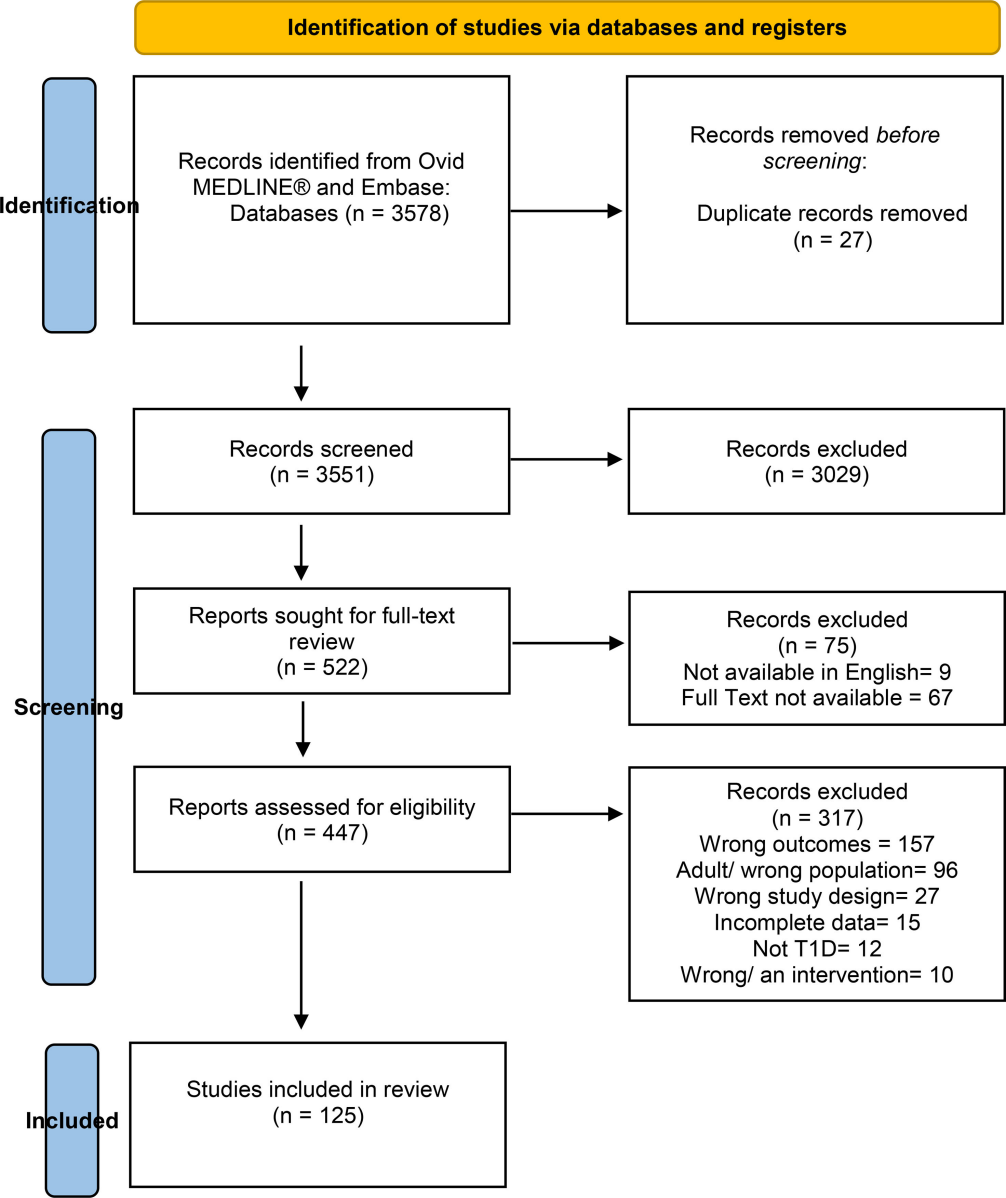
PRISMA flow diagram of study selection.

The searched yielded 3578 studies from which 3551 remained after duplicates were removed. In title and abstract screening, a further 3029 studies were removed. The full texts of the remaining 522 articles were reviewed. Following full-text review, a further 397 articles were excluded. The final review comprised 125 studies (**Figure 1**) encompassing 139 populations from 48 different countries. (The full list of papers included in this scoping review appears in **Supplementary Material 1**).

Most published studies (92 [75%]) were from high-income countries, followed by upper-middle income countries (13 [11%}), lower-middle income countries (13 [11%]) and then low-income countries (4 [3%]). Taiwan, where 3 studies originated, is unclassified by the World Bank into a country income group and was excluded from country income group analysis.

### New-Onset Type 1 Diabetes and iAb Prevalence

**Figure 2:**
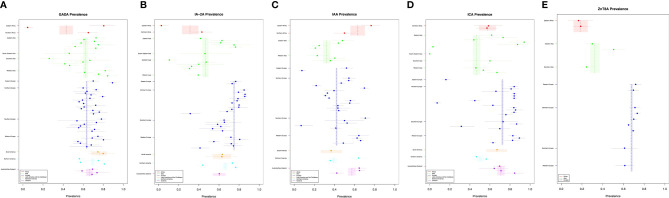
**(A–E)** New-onset T1DM and islet autoantibody prevalence.

The prevalence of each iAb in new-onset type 1 diabetes cases with 95% confidence intervals is displayed in relation to geographic location with weighted means for their respective continents (**Figures 2A–E**). Further data for numerator and denominator is included in the **Supplementary Material**.

Globally, IA-2A was the most prevalent iAb 0·714 (95% CI [0·71, 0·72]), followed by ICA 0·681 (95% CI [0·67, 0·69]), ZnT8A 0·654 (95% CI [0·64, 0·66]), GADA 0·636 (95% CI [0·63, 0·66]) and then IAA 0·424 (95% CI [0·42, 0·43]). GADA prevalence appeared to be similar across continents with the lower mean estimate for Africa driven by only one study while the other iAbs displayed more variability across geographic location. The weighted mean prevalence of GADA is highest in Latin American and lowest in Africa (**Figure 2A**). IA-2A prevalence is highest in Europe followed by Northern America, Latin America and the Caribbean, Oceania, Asia and Africa (**Figure 2B**). Converse to the other iAb, IAA was most prevalent in Africa followed by Oceania, Europe, Northern America, Latin America and the Caribbean, and Asia (**Figure 2C**). The weight mean prevalence of ICA was highest in Europe followed by Oceania, Latin America and the Caribbean, Africa, Northern America, and Asia (**Figure 2D**). Due to a paucity of data for ZnT8A, the weighted mean prevalence could only be calculated in three continents and was highest in Europe, followed by Asia and Africa (**Figure 2E**).

### Established Type 1 Diabetes and iAb Prevalence

**Figure 3:**
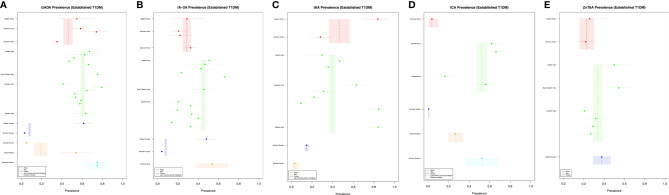
**(A–E)** Established T1DM and islet autoantibody prevalence.

The prevalence of each iAb in established type 1 diabetes cases with 95% confidence intervals are displayed in relation to geographic location with weighted means for their respective continents (**Figures 3A–E**). Further data for numerator and denominator is included in the **Supplementary Material**.

In established cases, GADA was the most prevalent iAb 0·407 (95% CI [0·39, 0·42]) followed by ZnT8A 0·322 (95% CI [0·29, 0·36]), IA-2A 0·302 (95% CI [0·29, 0·32]), IAA 0·258 (95% CI [0·24, 0·26]) and ICA 0·145 (95% CI [0·13, 0·16]).

GADA prevalence appeared to be similar across Europe, Asia and Africa, with the lower prevalence estimates found in Europe and Central and South America driven by one study respectively. IA-2A, ICA and IAA prevalence was most variable across settings. Converse to the prevalence of ZnT8A in new type 1 diabetes cases, ZnT8A established cases prevalence was similar across the three continents studied. The weighted man prevalence of GADA was highest Northern America followed by Asia, Africa, Latin American and the Caribbean and Europe (**Figure 3A**). The weighted mean prevalence of IA-2A was highest in Latin America and the Caribbean followed by Asia, Africa and Europe (**Figure 3B**). The prevalence of IAA was highest in Africa followed by Asia, and Latin America and the Caribbean (**Figure 3C**). ICA prevalence was highest in Northern America followed by Asia, Latin America and the Caribbean, Africa and Europe (**Figure 3D**). The prevalence of ZnT8A was highest in Europe followed by Asia and Africa (**Figure 3E**).

### Islet Autoantibody Positivity in Relation to the Number Tested

**Figure 4:**
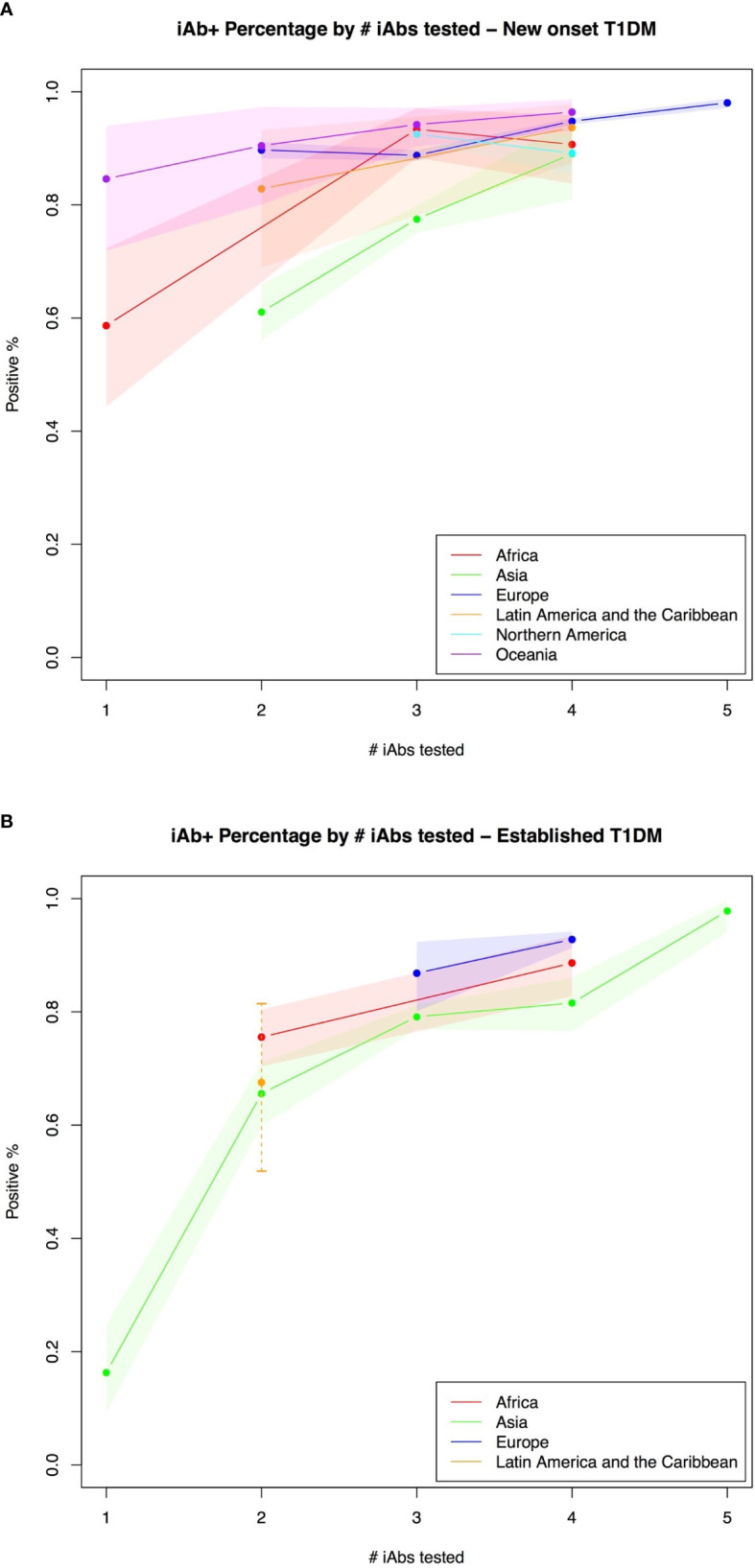
**(A, B)** Islet autoantibody positivity in relation to the number tested.

The mean positive percentage of iAb adjusted for the number tested was compared between continents. In new onset cases, for one iAb tested, the mean positive percentage for Oceania and Africa was 0·846 95% CI [0·72-0·94]) and 0·587 995% (CI [0·44-0·72]) respectively. When two iAb were tested, the highest mean positive percentage was found in Oceania 0·905 (95% CI [0·80-0·97]), followed by Europe 0·897 (95% CI [0·88-0·91]), Latin America and the Caribbean 0·829 (95% CI [0·69-0·93]) and then Asia 0·611 (95% CI [0·56-0·66]). When three iAb were tested, the highest mean positive percentage was found in Oceania 0·942 (95% CI [0·90-0·97]) followed by Africa 0·934 (95% CI [0·88-0·97]), Northern America 0·925 (95% CI [0·91-0·94]), Europe 0·888 (95% CI [0·88-0·90]) and Asia 0·775 (95% CI [0·75-0·80]). With four iAb tested, mean positive percentage was highest in Oceania 0·964 (95% CI [0·93-0·99]) followed by Europe 0·948 (95% CI [0·94-0·95]), Latin America and the Caribbean 0·937 (95% CI [0·87-0·98]), Africa 0·907 (95% CI [0·84-0·96]), Northern America 0·892 (95% CI [0·85-0·92]) and Asia 0·89 (95% CI [0·81-0·95]). For five iAb tested, only data for Europe exists, for which the mean positive percentage was 0·981 (95% CI [0.97-0.99]) (**Figure 4A**).

In established type 1 diabetes cases, for one iAb, only data for Asia exists where the mean positivity percentage was 0·163 (95% CI [0·09-0·25]). When two iAb were tested, the highest mean positivity percentage was found in Africa 0·755 (95% CI [0·70-0·80]) followed by Latin America and the Caribbean 0·676 (95% CI [0·52-0·81]) and Asia 0·656 (95% CI [0·60-0·71]). For three iAb tested, data exists for Europe 0·868 (95% CI [0·80-0·92]) and Asia 0·791 (95% CI [0·77-0·81]). When four iAb were tested, mean positivity percentage was highest in Europe 0·928 (95% CI [0·91-0·94]) followed by Africa 0·886 (95% CI [0·83-0·93]) and Asia 0·816 (95% CI [0·77-0·86]). For five iAb tested, only data for Asia exists, for which the mean positive percentage was 0·978 (95% CI [0·94-1·00])(**Figure 4B**).

## Discussion

To our knowledge, this is the first scoping review to synthesise the global evidence on the prevalence of iAb in children and adolescents aged 0-19 years with type 1 diabetes. We have analysed and present the global prevalence of iAb in children and adolescents with type 1 diabetes both for cases of new-onset and of a longer duration. Further, we have analysed and present the global differences in iAb positivity in relation to the number of iAb tested.

GADA prevalence appeared to be similar across settings with the lower mean estimate for Africa driven by only one study in which the controls had higher GADA percentage than the type 1 diabetic patients (27). Other iAbs display more variability across geographic regions: IA-2A, ICA and ZnT8A prevalence appears to be highest in Europe and lowest in Asia and Africa. Conversely, IAA prevalence appeared to be highest in Africa and Australia/New Zealand with lower prevalence seen in Europe, Asia and Oceania.

Studies tested for different numbers of iAbs, limiting the comparability between them. However, when controlling for the number of iAbs tested for, we found that the prevalence of iAb+ patients was similar by region, except for Asia, especially when fewer than 3 iAbs were tested for. This may be due to more variability in individual iAbs included in those studies. This highlights the importance of considering the iAb profiles included in each study. These results could inform future type 1 diabetes research given that prior studies have outlined that the number of iAb are more predictive of type 1 diabetes and its severity rather than iAb profile (19, 28). The global trends in type 1 diabetes iAb prevalence identified in this review echo prior literature (29–32).

This synthesis presents both opportunities and challenges. A more comprehensive understanding of iAb in type 1 diabetes and how it differs globally presents opportunities to improve type 1 diabetes diagnosis in children and adolescents before overt clinical symptoms emerge. Knowledge of the global prevalence and positivity of iAb could inform targeted type 1 diabetes-screening studies to reduce morbidity and mortality through closer monitoring, early detection and prompt management (13, 15).

Prior prospective studies trialling interventions while following iAb positive high risk individuals for developing type 1 diabetes have shown a very low DKA at overt type 1 diabetes onset (33) as well as lower HbA1c and insulin requirements at diagnosis. Although progression to overt type 1 diabetes from seroconversion is highly variable and the multiplicity of factors influencing the rate of progression are sub-optimally understood (19, 34), this encouraging precedent should encourage continued academic focus of iAb (25).

Numerous guidelines are in place on how to use iAb in clinical practice to improve the diagnosis of children and adolescents with type 1 diabetes. The Standards of Care in Diabetes, published by the America Diabetes Association, supports testing of iAb to diagnose type 1 diabetes as early as possible and begin management (35). The International Society for Paediatric and Adolescent Diabetes (ISPAD) clinical practice consensus guidelines outlines that both screening and intervention before the onset of symptoms of type 1 diabetes should be conducted within the context of defined research (18). The United Kingdom’s National Institute for Health and Care Excellence (NICE) type 1 diabetes diagnosis and management guidelines for children make one reference to iAb suggesting that iAb should not be measured to distinguish between type 1 and type 2 diabetes (36). In a subsection of this guideline titled ‘research recommendations’, there is no mention of iAb (36). The American Diabetes Association recommends that screening of iAb should currently only be done in a research context or can be offered to first-degree relatives of a proband with type 1 diabetes (37).

Given the rising global type 1 diabetes incidence and the potential ability of iAb to help in the prediction type 1 diabetes and subsequently confer the aforementioned clinical benefits and reduced morbidity and mortality, health policies and targeted healthcare interventions could now be developed to screen, diagnose and better manage, children and adolescents with type 1 diabetes (38). Knowledge about type 1 diabetes iAb prevalence could help to identify high-risk areas for type 1 diabetes and future cohort studies to elucidate factors that explain why some individuals with certain iAb profiles develop type 1 diabetes, progress at different rates and develop clinical sequelae. Finally, identification and quantification of the at-risk population of type 1 diabetes could help inform health policies and resource allocation to improve the capability of health systems to effectively and equitably screen, diagnose and manage children and adolescents with or at risk of developing type 1 diabetes.

While this study is important for providing a foundational knowledge of iAb prevalence, there are important gaps in iAb literature which this paper could not address such as iAb profile by age and the clinical manifestations (DKA, C-peptide levels, HbA1c) of respective iAb profiles. As such, challenges centre around the translation and utilisation of this data at various stages of the type 1 diabetes sequalae from prior to the onset of type 1 diabetes in screening to the acute and long-term management of type 1 diabetes. This scoping review has three primary limitations. First, there is a paucity of studies from low-income countries. In particular, there are only four studies from Africa (27, 39–41), a continent with an estimated population of 1.37 billion in 2021 of which 50.7% were aged 0-19 years (42). The majority (>90%) of studies analysed originated in high-income or upper-middle income countries. There is an urgent need for rigorous studies focussing on iAb in children and adolescents in low-income countries. Second, a significant proportion of iAb studies omitted years of study and data regarding positive islet autoantibody percentage overall (at least one iAb positive percentage), instead electing to prioritise data regarding respective iAb percentage. As prior literature has shown, data on the number of iAb may be of greater value than the individual iAb prevalence themselves (17–19). Future studies should follow minimum reporting requirements and outline both the years in which patients were diagnosed/detected, as well as the overall iAb positivity. The sparsity of such data meant we could not evaluate potential trends in iAb over time. Third, there is little sex and age disaggregated iAb data which is crucial to inform sex and age targeted healthcare interventions. Due to this, we were unable to determine if the iAb profile differs across age groups. This is especially crucial for iAb given that IAA is already known to be more prevalent in younger children (43). Without this data, it challenging to determine if differences exist for the remaining four iAb. Further, data presented of IAA prevalence in established type 1 diabetes should be interpreted with caution as antibodies may occur secondary to exogenous insulin administration and typically IAA should not be measured after two weeks of insulin treatment (44).

Since the discovery of iAb in 1974, iAb studies have flourished, moving from their discovery to their prevalence and clinical utility in screening both the general population and individuals at increased genetic risk for type 1 diabetes. The data on iAb by country, geographic region and country income group, could be useful to estimate better the global incidence of type 1 diabetes, particularly in LMICs where data are most scant, to develop health policies to more efficiently allocate health system resources, design targeted healthcare interventions to improve health outcomes for children and adolescents with type 1 diabetes. Future studies should build upon this foundational knowledge of iAb to address gaps in our understanding such as iAb profiles by age and their respective clinical manifestations.

## Data Availability Statement

The original contributions presented in the study are included in the article/**Supplementary Material**. Further inquiries can be directed to the corresponding author.

## Author Contributions

The article was conceptualized by RA and C-LR, who secured support from CDIC and set up a writing team. Review, data curation and formal analysis were performed by CR and ZW with second reviews conducted by AG and MO. All authors contributed equally to manuscript writing, revisions, and final approval. All authors take full responsibility for the contents of the article.

## Conflict of Interest

The authors declare that the research was conducted in the absence of any commercial or financial relationships that could be construed as a potential conflict of interest.

## Publisher’s Note

All claims expressed in this article are solely those of the authors and do not necessarily represent those of their affiliated organizations, or those of the publisher, the editors and the reviewers. Any product that may be evaluated in this article, or claim that may be made by its manufacturer, is not guaranteed or endorsed by the publisher.
