# Supplementary material for: The Prevalence of Islet Autoantibodies in Children and Adolescents With Type 1 Diabetes Mellitus: A Global Scoping Review
Source: Front Endocrinol (Lausanne). 2022 Feb 3;13:815703. doi: 10.3389/fendo.2022.815703 (PMC8851309; doi:10.3389/fendo.2022.815703)
Supplement: Supplementary file 1 [file DataSheet_1.docx]

**Supplementary Files**

**Contents**

[Search strategy concepts 2](#_Toc82420010)

[Table 1: Search strategy concepts 2](#_Toc82420011)

[Supplementary Tables 3](#_Toc82420012)

[Table 1: GADA New Cases 3](#_Toc82420013)

[Table 2: GADA Established Cases 3](#_Toc82420014)

[Table 3: IA-2A New Cases 3](#_Toc82420015)

[Table 4: IA-2A Established Cases 3](#_Toc82420016)

[Table 5: IAA New Cases 3](#_Toc82420017)

[Table 6: IAA Established Cases 4](#_Toc82420018)

[Table 7: ICA New Cases 4](#_Toc82420019)

[Table 8: ICA Established Cases 4](#_Toc82420020)

[Table 9: ZnT8A New Cases 4](#_Toc82420021)

[Table 10: ZnT8A Established Cases 5](#_Toc82420022)

[Scoping review articles 6](#_Toc82420023)

#

# Search strategy concepts

## Table 1: Search strategy concepts

|  | **Type 1 Diabetes Mellitus** | **Antibodies** | **Population** | **Publication date** |
| --- | --- | --- | --- | --- |
| Controlled vocabulary Terms  [MeSH] | "Diabetes Mellitus, Type 1" | "Antibodies, iselt" | "Infant"  "Child"  "Adolescent" | 1990[pdat]: 2021[pdat] |
| Synonyms  (tiab) | Diabetes  Diabetic  AND  Type 1  OR  Type I  OR  Childhood  OR  Juvenile onset  OR  Autoimmune  OR  Auto immune  OR  Insulin dependant | antibody  OR  Antibodies  AND  Islet  OR  islet cell  OR  Islet-antibody  OR  islet-antibodies | Neonatal  OR  Infant*  OR  Infancy  OR  Preschool  OR  Child*  OR  Juvenile*  OR  Adolescen*  OR  Puberty  OR  Pubertal  OR  Teen*  OR  Pediatric  OR  Paediatric |  |
| **Combination Search:**  ("Diabetes Mellitus, Type 1"[Mesh] OR (diabetes[tiab] OR diabetic[tiab]) AND (type 1[tiab] OR type I[tiab] OR childhood[tiab] OR juvenile onset[tiab] OR autoimmune[tiab] OR auto immune[tiab] OR insulin-dependent[tiab])) AND (“Autoantibodies, Islet” [Mesh] OR (antibody[tiab] OR antibodies[tiab] AND (islet[tiab] OR islet cell[tiab] OR islet-antibody[tiab] OR islet-antibodies) AND ("Infant"[Mesh] OR "Child"[Mesh] OR "Adolescent"[Mesh] OR neonatal[tiab] OR infant*[tiab] OR infancy[tiab] OR preschool[tiab] OR child*[tiab] OR juvenile*[tiab] OR adolescen*[tiab] OR puberty[tiab] OR pubertal[tiab] OR teen*[tiab] OR pediatric[tiab] OR paediatric[tiab]) AND (1990[pdat] : 2021[pdat]) | | | | |

#

# Supplementary Tables

**iAb mean weight prevalence, 95% CI and Numerator (x)/ denominator (N)**

## Table 1: GADA New Cases

| **Area** | **Mean** | **lb** | **ub** | **x** | **N** |
| --- | --- | --- | --- | --- | --- |
| Africa | 0.42995169 | 0.36338486 | 0.49780088 | 89 | 207 |
| Asia | 0.62372065 | 0.60029211 | 0.64686699 | 1036 | 1661 |
| Europe | 0.63568773 | 0.62727944 | 0.64405536 | 8037 | 12643 |
| Latin America and the Caribbean | 0.76315789 | 0.68132088 | 0.83625409 | 87 | 114 |
| Northern America | 0.6963807 | 0.67281148 | 0.71945124 | 1039 | 1492 |
| Oceania | 0.66243655 | 0.62906075 | 0.69503134 | 522 | 788 |
| Global | 0.63945578 | 0.63220236 | 0.64667795 | 10810 | 16905 |

## Table 2: GADA Established Cases

| **Area** | **mean** | **lb** | **ub** | **x** | **N** |
| --- | --- | --- | --- | --- | --- |
| Africa | 0.4612069 | 0.41607755 | 0.50665303 | 214 | 464 |
| Asia | 0.60740741 | 0.58604559 | 0.62856827 | 1230 | 2025 |
| Europe | 0.08163265 | 0.06749448 | 0.09696832 | 108 | 1323 |
| Latin America and the Caribbean | 0.18656716 | 0.12549339 | 0.25649344 | 25 | 134 |
| Northern America | 0.75 | 0.6501743 | 0.83799407 | 60 | 80 |
| Global | 0.40660705 | 0.39148078 | 0.42182122 | 1637 | 4026 |

## Table 3: IA-2A New Cases

| **Area** | **mean** | **lb** | **ub** | **x** | **N** |
| --- | --- | --- | --- | --- | --- |
| Africa | 0.31147541 | 0.23274451 | 0.39606092 | 38 | 122 |
| Asia | 0.46590909 | 0.43590385 | 0.49603665 | 492 | 1056 |
| Europe | 0.74921921 | 0.7413745 | 0.75698422 | 8876 | 11847 |
| Latin America and the Caribbean | 0.63157895 | 0.54141496 | 0.71736899 | 72 | 114 |
| Northern America | 0.66184573 | 0.63731389 | 0.68595527 | 961 | 1452 |
| Oceania | 0.60344828 | 0.53985355 | 0.66535336 | 140 | 232 |
| Global | 0.71368819 | 0.70638424 | 0.72093752 | 10579 | 14823 |

## Table 4: IA-2A Established Cases

| **Area** | **mean** | **lb** | **ub** | **x** | **N** |
| --- | --- | --- | --- | --- | --- |
| Africa | 0.28602151 | 0.24588918 | 0.3278972 | 133 | 465 |
| Asia | 0.45071138 | 0.42878389 | 0.47273376 | 887 | 1968 |
| Europe | 0.07936508 | 0.06541867 | 0.09451546 | 105 | 1323 |
| Latin America and the Caribbean | 0.54054054 | 0.38097679 | 0.69594941 | 20 | 37 |
| Global | 0.30187187 | 0.2873643 | 0.31657734 | 1145 | 3793 |

## Table 5: IAA New Cases

| **area** | **mean** | **lb** | **ub** | **x** | **N** |
| --- | --- | --- | --- | --- | --- |
| Africa | 0.63157895 | 0.55813266 | 0.70210948 | 108 | 171 |
| Asia | 0.32242991 | 0.28684113 | 0.3590666 | 207 | 642 |
| Europe | 0.42106494 | 0.41253281 | 0.42962039 | 5401 | 12827 |
| Latin America and the Caribbean | 0.36708861 | 0.26497731 | 0.47557597 | 29 | 79 |
| Northern America | 0.39277389 | 0.36036068 | 0.4256606 | 337 | 858 |
| Oceania | 0.57250755 | 0.53465545 | 0.60994466 | 379 | 662 |
| Global | 0.42397795 | 0.41614154 | 0.43183326 | 6461 | 15239 |

## Table 6: IAA Established Cases

| **Area** | **mean** | **lb** | **ub** | **x** | **N** |
| --- | --- | --- | --- | --- | --- |
| Africa | 0.46315789 | 0.3643739 | 0.5634119 | 44 | 95 |
| Asia | 0.39695431 | 0.3666205 | 0.42768449 | 391 | 985 |
| Europe | 0.14722911 | 0.127826 | 0.16773749 | 178 | 1209 |
| Latin America and the Caribbean | 0.03092784 | 0.00649145 | 0.07323683 | 3 | 97 |
| Global | 0.25817267 | 0.24081089 | 0.27591844 | 616 | 2386 |

## Table 7: ICA New Cases

| **Area** | **mean** | **lb** | **ub** | **x** | **N** |
| --- | --- | --- | --- | --- | --- |
| Africa | 0.57575758 | 0.49081718 | 0.65852287 | 76 | 132 |
| Asia | 0.46751412 | 0.43088584 | 0.50431626 | 331 | 708 |
| Europe | 0.72339768 | 0.71243823 | 0.73422642 | 4684 | 6475 |
| Latin America and the Caribbean | 0.67088608 | 0.56416135 | 0.76941476 | 53 | 79 |
| Northern America | 0.50142857 | 0.46442261 | 0.5384268 | 351 | 700 |
| Oceania | 0.70890411 | 0.6714331 | 0.74501989 | 414 | 584 |
| Global | 0.68091726 | 0.67107161 | 0.69068393 | 5909 | 8678 |

## Table 8: ICA Established Cases

| **Area** | **mean** | **lb** | **ub** | **x** | **N** |
| --- | --- | --- | --- | --- | --- |
| Africa | 0.03703704 | 0.00460311 | 0.10070153 | 2 | 54 |
| Asia | 0.52254642 | 0.47210791 | 0.57275831 | 197 | 377 |
| Europe | 0.00661704 | 0.00286334 | 0.01190265 | 8 | 1209 |
| Latin America and the Caribbean | 0.26428571 | 0.19485383 | 0.34009493 | 37 | 140 |
| Northern America | 0.52777778 | 0.36645802 | 0.68617149 | 19 | 36 |
| Global | 0.14482379 | 0.1290178 | 0.16137066 | 263 | 1816 |

## Table 9: ZnT8A New Cases

| **area** | **mean** | **lb** | **ub** | **x** | **N** |
| --- | --- | --- | --- | --- | --- |
| Africa | 0.18181818 | 0.11860642 | 0.25498003 | 22 | 121 |
| Asia | 0.3241206 | 0.2790628 | 0.37085269 | 129 | 398 |
| Europe | 0.6793299 | 0.66877974 | 0.68979045 | 5150 | 7581 |
| Global | 0.65444444 | 0.64405307 | 0.66476358 | 5301 | 8100 |

## Table 10: ZnT8A Established Cases

| **area** | **mean** | **lb** | **ub** | **x** | **N** |
| --- | --- | --- | --- | --- | --- |
| Africa | 0.22727273 | 0.16015749 | 0.30221133 | 30 | 132 |
| Asia | 0.33626374 | 0.29360819 | 0.38028272 | 153 | 455 |
| Europe | 0.37719298 | 0.29083362 | 0.4676348 | 43 | 114 |
| Global | 0.32239658 | 0.2883144 | 0.35743865 | 226 | 701 |

#

# Scoping review reference list for references cited solely in Supplementary Material

1. Gesualdo PD, Bautista KA, Waugh KC, Yu L, Norris JM, Rewers MJ, et al. Feasibility of screening for T1D and celiac disease in a pediatric clinic setting. Pediatr Diabetes [Internet]. 2016 Sep;17(6):441–8. Available from: https://onlinelibrary.wiley.com/doi/10.1111/pedi.12301

2. Gowiñska-Olszewska B, Michalak J, Luczyñski W, Del Pilar Larosa M, Chen S, Furmaniak J, et al. Organ-specific autoimmunity in relation to clinicalR characteristics in children with long-lasting type 1 diabetes. J Pediatr Endocrinol Metab. 2016;29(6):647–56.

3. Kim SY, Kim EY, Hwang JS. Clinical characteristics and laboratory findings of children and adolescents with diabetes. Int J Pediatr Endocrinol. 2013;2013(S1):2013.

4. Kimpimäki T, Kulmala P, Savola K, Vähäsalo P, Reijonen H, Ilonen J, et al. Disease-associated autoantibodies as surrogate markers of type 1 diabetes in young children at increased genetic risk. J Clin Endocrinol Metab. 2000;85(3):1126–32.

5. Kimpimäki T, Kulmala P, Savola K, Kupila A, Korhonen S, Simell T, et al. Natural history of β-cell autoimmunity in young children with increased genetic susceptibility to type 1 diabetes recruited from the general population. J Clin Endocrinol Metab. 2002;87(10):4572–9.

6. Knip M, Karjalainen J, Akerblom HK. Islet cell antibodies are less predictive of IDDM among unaffected children in the general population than in sibs of children with diabetes. The Childhood Diabetes in Finland Study Group. Diabetes Care [Internet]. 1998 Oct;21(10):1670–3. Available from: http://www.ncbi.nlm.nih.gov/pubmed/9773728

7. Komulainen J, Kulmala P, Savola K, Lounamaa R, Ilonen J, Reijonen H, et al. Clinical, autoimmune, and genetic characteristics of very young children with type 1 diabetes. Childhood Diabetes in Finland (DiMe) Study Group. Diabetes Care [Internet]. 1999 Dec 1;22(12):1950–5. Available from: http://care.diabetesjournals.org/cgi/doi/10.2337/diacare.22.12.1950

8. Hagopian WA, Sanjeevi CB, Kockum I, Landin-Olsson M, Karlsen AE, Sundkvist G, et al. Glutamate decarboxylase-, insulin-, and islet cell-antibodies and HLA typing to detect diabetes in a general population-based study of Swedish children. J Clin Invest. 1995;95(4):1505–11.

9. Hawa M, Rowe R, Lan MS, Notkins AL, Pozzilli P, Christie MR, et al. Value of antibodies to islet protein tyrosine phosphatase-like molecule in predicting type 1 diabetes. Diabetes. 1997;46(8):1270–5.

10. Hermann R, Soltész G. Prevalence and HLA association of GAD65 antibodies in Hungarian schoolchildren. Hum Immunol. 2003;64(1):152–5.

11. Holmberg H, Vaarala O, Sadauskaite - Kuehne V, Ilonen J, Padaiga Ž, Ludvigsson J. Higher prevalence of autoantibodies to insulin and GAD65 in Swedish compared to Lithuanian children with type 1 diabetes. Diabetes Res Clin Pract. 2006;72(3):308–14.

12. Juusola M, Parkkola A, Härkönen T, Siljander H, Ilonen J, Åkerblom HK, et al. Positivity for Zinc Transporter 8 Autoantibodies at Diagnosis Is Subsequently Associated with Reduced b-Cell Function and Higher Exogenous Insulin Requirement in Children and Adolescents with Type 1 Diabetes. Diabetes Care. 2016;39(1):118–21.

13. Karagüzel G, Şimşek S, Deǧer O, Ökten A. Screening of diabetes, thyroid, and celiac diseases-related autoantibodies in a sample of Turkish children with type 1 diabetes and their siblings. Diabetes Res Clin Pract. 2008;80(2):238–43.

14. Kawasaki E, Oikawa Y, Okada A, Kanatsuna N, Kawamura T, Kikuchi T, et al. Different interaction of onset age and duration of type 1 diabetes on the dynamics of autoantibodies to insulinoma-associated antigen-2 and zinc transporter 8. J Diabetes Investig. 2021;12(4):510–5.

15. Kelly MA, Alvi NS, Croft NJ, Mijovic CH, Bottazzo GF, Barnett AH. Genetic and immunological characteristics of type I diabetes mellitus in an Indo-Aryan population. Diabetologia. 2000;43(4):450–6.

16. Kondrashova A, Viskari H, Kulmala P, Romanov A, Ilonen J, Hyöty H, et al. Signs of β-cell autoimmunity in nondiabetic schoolchildren: A comparison between Russian Karelia with a low incidence of type 1 diabetes and Finland with a high incidence rate. Diabetes Care. 2007;30(1):95–100.

17. Kordonouri O, Hartmann R, Grüters-Kieslich A, Knip M, Danne T. Age-specific levels of diabetes-related GAD and IA-2 antibodies in healthy children and adults. J Pediatr Endocrinol Metab. 2002;15(1):47–52.

18. Lévy-Marchal C, Dubois F, Noël M, Tichet J, Czernichow P. Immunogenetic determinants and prediction of IDDM in French schoolchildren. Diabetes [Internet]. 1995 Sep;44(9):1029–32. Available from: http://www.ncbi.nlm.nih.gov/pubmed/7657024

19. Libman IM, Pietropaolo M, Trucco M, Dorman JS, LaPorte RE, Becker D. Islet cell autoimmunity in white and black children and adolescents with IDDM. Diabetes Care [Internet]. 1998 Nov;21(11):1824–7. Available from: http://www.ncbi.nlm.nih.gov/pubmed/9802728

20. Lindberg B, Ivarsson SA, Landin-Olsson M, Sundkvist G, Svanberg L, Lernmark Å. Islet autoantibodies in cord blood from children who developed type I (insulin-dependent) diabetes mellitus before 15 years of age. Diabetologia. 1999;42(2):181–7.

21. Lindberg B, Carlsson A, Ericsson UB, Kockum I, Lernmark Å, Landin-Olsson M, et al. Prevalence of β-cell and thyroid autoantibody positivity in schoolchildren during three-year follow-up. Autoimmunity. 1999;31(3):175–85.

22. Long AE, Gillespie KM, Rokni S, Bingley PJ, Williams AJK. Rising incidence of type 1 diabetes is associated with altered immunophenotype at diagnosis. Diabetes. 2012;61(3):683–6.

23. Ludvigsson J, Hellström S. Autoantibodies in relation to residual insulin secretion in children with IDDM. Diabetes Res Clin Pract. 1997;35(2–3):81–9.

24. Luo S, Li X, Huang G, Xie Z, Xiang Y, Dai Z, et al. Distinct two different ages associated with clinical profiles of acute onset type 1 diabetes in Chinese patients. Diabetes Metab Res Rev. 2020;36(2):1–8.

25. Korneva KG, Strongin LG, Kolbasina E V., Budylina M V., Makeeva N V., Zagainov VE. Diagnostic capabilities of islet autoantibodies in children with new-onset type 1 diabetes mellitus and healthy siblings. Sovrem Tehnol v Med. 2020;12(6):29–35.

26. Krischer JP, Liu X, Vehik K, Akolkar B, Hagopian WA, Rewers MJ, et al. Predicting islet cell autoimmunity and type 1 diabetes: An 8-year teddy study progress report. Diabetes Care. 2019;42(6):1051–60.

27. Kulmala P, Savola K, Petersen JS, Vähäsalo P, Karjalainen J, Löppönen T, et al. Prediction of insulin-dependent diabetes mellitus in siblings of children with diabetes: A population-based study. J Clin Invest. 1998;101(2):327–36.

28. LaGasse JM, Brantley MS, Leech NJ, Rowe RE, Monks S, Palmer JP, et al. Successful Prospective Prediction of Type 1 Diabetes in Schoolchildren Through. Diabetes Care. 2002;25(3):505–11.

29. Landin-Olsson M, Palmer JP, Lernmark A, Blom L, Sundkvist G, Nyström L, et al. Predictive value of islet cell and insulin autoantibodies for type 1 (insulin-dependent) diabetes mellitus in a population-based study of newly-diagnosed diabetic and matched control children. Diabetologia [Internet]. 1992 Nov;35(11):1068–73. Available from: http://www.ncbi.nlm.nih.gov/pubmed/1473617

30. Lee YS, Ng WY, Thai AC, Lui KF, Loke KY. Prevalence of ICA and GAD antibodies at initial presentation of type 1 diabetes mellitus in Singapore children. J Pediatr Endocrinol Metab. 2001;14(6):767–72.

31. Leech NJ, Kitabchi AE, Gaur LK, Hagopian WA, Hansen J, Burghen GA, et al. Genetic and immunological markers of insulin dependent diabetes in black americans. Autoimmunity. 1995;22(1):27–32.

32. Lévy-Marchal C, Tichet J, Fajardy I, Gu XF, Dubois F, Czernichow P. Islet cell antibodies in normal French schoolchildren. Diabetologia [Internet]. 1992 Jun;35(6):577–82. Available from: http://www.ncbi.nlm.nih.gov/pubmed/1612232

33. Majeed NA, Shiruhana SA, Maniam J, Eigenmann CA, Siyan A, Ogle GD. Incidence, prevalence and mortality of diabetes in children and adolescents aged under 20 years in the Republic of Maldives. J Paediatr Child Health [Internet]. 2020 May 23;56(5):746–50. Available from: https://onlinelibrary.wiley.com/doi/10.1111/jpc.14726

34. Marčiulionyte D, Williams AJK, Bingley PJ, Urbonaite B, Gale EAM. A comparison of the prevalence of islet autoantibodies in children from two countries with differing incidence of diabetes. Diabetologia. 2001;44(1):16–21.

35. Park Y, Lee H, Takino H, Abiru N, Kawasaki E, Eisenbarth GS. Evaluation of the efficacy of the combination of multiple autoantibodies to islet-specific antigens in Korean type 1 diabetic patients. Acta Diabetol. 2001;38(1):51–6.

36. Perchard R, MacDonald D, Say J, Pitts J, Pye S, Allgrove J, et al. Islet autoantibody status in a multi-ethnic UK clinic cohort of children presenting with diabetes. Arch Dis Child. 2015;100(4):348–52.

37. Petruzelkova L, Ananieva-Jordanova R, Vcelakova J, Vesely Z, Stechova K, Lebl J, et al. The dynamic changes of zinc transporter 8 autoantibodies in Czech children from the onset of Type 1 diabetes mellitus. Diabet Med. 2014;31(2):165–71.

38. Ponsonby AL, Pezic A, Cameron FJ, Rodda C, Ellis JA, Kemp AS, et al. Phenotypic and environmental factors associated with elevated autoantibodies at clinical onset of paediatric type 1 diabetes mellitus. Results Immunol [Internet]. 2012;2:125–31. Available from: http://dx.doi.org/10.1016/j.rinim.2012.06.002

39. Pozzilli P, Visalli N, Buzzetti R, Cavallo MG, Marietti G, Hawa M, et al. Metabolic and immune parameters at clinical onset of insulin-dependent diabetes: A population-based study. Metabolism. 1998;47(10):1205–10.

40. Premawardhana LDKE, Wijeyaratne CN, Chen S, Wijesuriya M, Illangasekera U, Brooking H, et al. Islet cell, thyroid, adrenal and celiac disease related autoantibodies in patients with Type 1 diabetes from Sri Lanka. J Endocrinol Invest [Internet]. 2006 Dec;29(11):968–74. Available from: http://www.ncbi.nlm.nih.gov/pubmed/17259793

41. Raab J, Haupt F, Scholz M, Matzke C, Warncke K, Lange K, et al. Capillary blood islet autoantibody screening for identifying pre-type 1 diabetes in the general population: design and initial results of the Fr1da study. BMJ Open [Internet]. 2016 May 18;6(5):e011144. Available from: https://bmjopen.bmj.com/lookup/doi/10.1136/bmjopen-2016-011144

42. Tuomilehto-wolf EVA, Akerblom HK. Glutamic Antibodies in Relation and Genetic Risk Markers. J Clin Endocrinol Metab. 1996;81(7):2455–9.

43. Marita AR, Rane S, Mokal RA, Nair SR, Irani A. Autoantibodies against GAD65 and IA-2 in recently diagnosed Type 1 diabetic children from Western India. Diabet Med [Internet]. 2004 Aug;21(8):956–7. Available from: http://www.ncbi.nlm.nih.gov/pubmed/15270809

44. Mimura G, Kida K, Murakami K. A multicenter study on HLA and autoimmunity in Japanese patients with early-onset insulin-dependent diabetes mellitus (IDDM): the JDS Study. Diabetes Res Clin Pract [Internet]. 1994 Oct;24 Suppl:S75-81. Available from: http://www.ncbi.nlm.nih.gov/pubmed/7859638

45. Mortensen HB, Swift PG, Holl RW, Hougaard P, Hansen L, Bjoerndalen H, et al. Multinational study in children and adolescents with newly diagnosed type 1 diabetes: Association of age, ketoacidosis, HLA status, and autoantibodies on residual beta-cell function and glycemic control 12 months after diagnosis. Pediatr Diabetes. 2010;11(4):218–26.

46. Ng WY, Lee YS, Todd AL, Lui KF, Loke KY, Thai AC. Tyrosine phosphatase-like protein (IA-2) and glutamic acid decarboxylase (GAD65) autoantibodies: A study of Chinese patients with diabetes mellitus. Autoimmunity. 2002;35(2):119–24.

47. Nieto J, Castillo B, Astudillo M, Tosur M, Balasubramanyam A, Pietropaolo M, et al. Islet autoantibody types mark differential clinical characteristics at diagnosis of pediatric type 1 diabetes. Pediatr Diabetes. 2021;22(6):882–8.

48. Padoa CJ, Rheeder P, Pirie FJ, Motala AA, van Dyk JC, Crowther NJ. Identification of a subgroup of black South Africans with type 1 diabetes who are older at diagnosis but have lower levels of glutamic acid decarboxylase and islet antigen 2 autoantibodies. Diabet Med. 2020;37(12):2067–74.

49. Pan S, Wu T, Shi X, Xie Z, Huang G, Zhou Z. Organ-specific autoantibodies in Chinese patients newly diagnosed with type 1 diabetes mellitus. Endocr J. 2020;67(7):793–802.

50. Pardini VC, Mourão DM, Nascimento PD, Vívolo MA, Ferreira SRG, Pardini H. Frequency of islet cell autoantibodies (IA-2 and GAD) in young Brazilian type 1 diabetes patients. Brazilian J Med Biol Res. 1999;32(10):1195–8.

51. Sabbah E, Savola K, Ebeling T, Kulmala P, Vähäsalo P, Ilonen J, et al. Genetic, autoimmune, and clinical characteristics of childhood- and adult-onset type 1 diabetes. Diabetes Care [Internet]. 2000 Sep;23(9):1326–32. Available from: http://www.ncbi.nlm.nih.gov/pubmed/10977027

52. Samuelsson UU, Sundkvist GG, Borg HH, Fernlund PP, Ludvigsson JJ. Islet autoantibodies in the prediction of diabetes in school children. Diabetes Res Clin Pract. 2001;51(1):51–7.

53. Siljander HT, Veijola R, Reunanen A, Virtanen SM, Åkerblom HK, Knip M. Prediction of type 1 diabetes among siblings of affected children and in the general population. Diabetologia. 2007;50(11):2272–5.

54. Simmons KM, Youngkin E, Alkanani A, Miao D, McDaniel K, Yu L, et al. Screening children for type 1 diabetes-associated antibodies at community health fairs. Pediatr Diabetes. 2019;20(7):909–14.

55. Sorensen JS, Vaziri-Sani F, Maziarz M, Kristensen K, Ellerman A, Breslow N, et al. Islet autoantibodies and residual beta cell function in type 1 diabetes children followed for 3-6 years. Diabetes Res Clin Pract. 2012;96(2):204–10.

56. Steck AK, Vehik K, Bonifacio E, Lernmark A, Ziegler AG, Hagopian WA, et al. Predictors of progression from the appearance of islet autoantibodies to early childhood diabetes: The Environmental Determinants of Diabetes in the Young (TEDDY). Diabetes Care. 2015;38(5):808–13.

57. Strebelow M, Schlosser M, Ziegler B, Rjasanowski I, Ziegler M. Karlsburg Type I diabetes risk study of a general population: Frequencies and interactions of the four major Type I diabetes-associated autoantibodies studied in 9419 schoolchildren. Diabetologia. 1999;42(6):661–70.

58. Trisorus C, Aroonparkmongkol S, Kongmanas HB, Sahakitrungruang T. Prevalence of islet autoantibodies in Thai juvenile-onset type 1 diabetes. Pediatr Int. 2018;60(11):1002–7.

59. Tung YC, Chen MH, Lee CT, Tsai WY. β-Cell Autoantibodies and Their Function in Taiwanese Children With Type 1 Diabetes Mellitus. J Formos Med Assoc [Internet]. 2009;108(11):856–61. Available from: http://dx.doi.org/10.1016/S0929-6646(09)60417-4

60. Urakami T, Miyamoto Y, Matsunaga H, Owada M, Kitagawa T. Serial changes in the prevalence of islet cell antibodies and islet cell antibody titer in children with IDDM of abrupt or slow onset. Diabetes Care. 1995;18(8):1095–9.

61. Sabbah E, Savola K, Kulmala P, Veijola R, Vähäsalo P, Karjalainen J, et al. Diabetes-Associated Autoantibodies in Relation to Clinical Characteristics and Natural Course in Children with Newly Diagnosed Type 1 Diabetes 1. J Clin Endocrinol Metab [Internet]. 1999 May;84(5):1534–9. Available from: http://www.ncbi.nlm.nih.gov/pubmed/10323375

62. Savola K, Bonifacio E, Sabbah E, Kulmala P, Vähäsalo P, Karjalainen J, et al. IA-2 antibodies - A sensitive marker of IDDM with clinical onset in childhood and adolescence. Diabetologia. 1998;41(4):424–9.

63. Savola K, Sabbah E, Kulmala P, Vähäsalo P, Ilonen J, Knip M. Autoantibodies associated with Type I diabetes mellitus persist after diagnosis in children. Diabetologia. 1998;41(11):1293–7.

64. Schatz D, Krischer J, Horne G, Riley W, Spillar R, Silverstein J, et al. Islet cell antibodies predict insulin-dependent diabetes in United States school age children as powerfully as in unaffected relatives. J Clin Invest. 1994;93(6):2403–7.

65. Scheinin T, Minh NNT, Tuomi T, Miettinen A, Kontiainen S. Islet cell and glutamic acid decarboxylase antibodies and heat-shock protein 65 responses in children with newly diagnosed insulin-dependent diabetes mellitus. Immunol Lett. 1996;49(1–2):123–6.

66. Sera Y, Kawasaki E, Abiru N, Ozaki M, Abe T, Takino H, et al. Autoantibodies to multiple islet autoantigens in patients with abrupt onset type I diabetes and diabetes diagnosed with urinary glucose screening. J Autoimmun. 1999;13(2):257–65.

67. Wang J, Miao D, Babu S, Yu J, Barker J, Klingensmith G, et al. Prevalence of autoantibody-negative diabetes is not rare at all ages and increases with older age and obesity. J Clin Endocrinol Metab [Internet]. 2007 Jan;92(1):88–92. Available from: http://www.ncbi.nlm.nih.gov/pubmed/17062766

68. Shivaprasad C, Mittal R, Dharmalingam M, Kumar P. Zinc transporter-8 autoantibodies can replace IA-2 autoantibodies as a serological marker for juvenile onset type 1 diabetes in India. Indian J Endocrinol Metab. 2014;18(3):345–9.

69. Sunni M, Noble JA, Yu L, Mahamed Z, Lane JA, Dhunkal AM, et al. Predominance of DR3 in Somali children with type 1 diabetes in the twin cities, Minnesota. Pediatr Diabetes [Internet]. 2017 Mar;18(2):136–42. Available from: https://onlinelibrary.wiley.com/doi/10.1111/pedi.12369

70. Vähäsalo P, Knip M, Karjalainen J, Tuomilehto-Wolf E, Lounamaa R, Åkerblom HK. Islet cell-specific autoantibodies in children with insulin-dependent diabetes mellitus and their siblings at clinical manifestation of the disease. Eur J Endocrinol. 1996;135(6):689–95.

71. Zanone MM, Catalfamo E, Pietropaolo SL, Rabbone I, Sacchetti C, Cerutti F, et al. Glutamic acid decarboxylase and ICA512/IA-2 autoantibodies as disease markers and relationship to residual beta-cell function and glycemic control in young type 1 diabetic patients. Metabolism. 2003;52(1):25–9.

72. VANDEWALLE CL, MARINA I. COECKELBERGHS IHDL, CAJU MV Du, SCHUIT FC, DANIEL G. PIPELEERS, GORUS FK. Epidemiology, Clinical Aspects, and Biology of IDDM Patients Under Age 4 0 Years. Diabetes Care. 1997;20(10):1556–61.

73. Verge CF, Howard NJ, Rowley MJ, Mackay IR, Zimmet PZ, Egan M, et al. Anti-glutamate decarboxylase and other antibodies at the onset of childhood IDDM: a population-based study. Diabetologia [Internet]. 1994 Nov;37(11):1113–20. Available from: http://www.ncbi.nlm.nih.gov/pubmed/7867883

74. Verkauskiene R, Danyte E, Dobrovolskiene R, Stankute I, Simoniene D, Razanskaite-Virbickiene D, et al. The course of diabetes in children, adolescents and young adults: Does the autoimmunity status matter? BMC Endocr Disord [Internet]. 2016;16(1):1–13. Available from: http://dx.doi.org/10.1186/s12902-016-0145-3

75. Vipin VP, Zaidi G, Watson K, G Colman P, Prakash S, Agrawal S, et al. High prevalence of idiopathic (islet antibody-negative) type 1 diabetes among Indian children and adolescents. Pediatr Diabetes [Internet]. 2021;22(1):47–51. Available from: http://www.ncbi.nlm.nih.gov/pubmed/32558116

76. Yaghootkar H, Abbasi F, Ghaemi N, Rabbani A, Wakeling MN, Eshraghi P, et al. Type 1 diabetes genetic risk score discriminates between monogenic and Type 1 diabetes in children diagnosed at the age of <5 years in the Iranian population. Diabet Med. 2019;36(12):1694–702.

77. Yamada H, Uchigata Y, Kawasaki E, Matsuura N, Otani T, Sato A, et al. Onset age-dependent variations of three islet specific autoantibodies in Japanese IDDM patients. Diabetes Res Clin Pract. 1998;39(3):211–7.

78. Yu J, Shin CH, Yang SW, Park MH, Eisenbarth GS. Analysis of children with type 1 diabetes in Korea: High prevalence of specific anti-islet autoantibodies, immunogenetic similarities to Western populations with “unique” haplotypes, and lack of discrimination by aspartic acid at position 57 of DQB. Clin Immunol. 2004;113(3):318–25.

79. Zabeen B, Govender D, Hassan Z, Noble JA, Lane JA, Mack SJ, et al. Clinical features, biochemistry and HLA-DRB1 status in children and adolescents with diabetes in Dhaka, Bangladesh. Diabetes Res Clin Pract [Internet]. 2019 Dec;158(1):107894. Available from: http://www.ncbi.nlm.nih.gov/pubmed/31669629

80. Abdullah MA, Bahakim H, Rab MOG Al, Halim K, Salman H, Abanamy A. Antithyroid and Other Organ-specific Antibodies in Saudi Arab Diabetic and Normal Children. Diabet Med [Internet]. 1990 Jan;7(1):50–2. Available from: https://onlinelibrary.wiley.com/doi/10.1111/j.1464-5491.1990.tb01307.x

81. Ahmad J, Siddiqui MS, Ahmed F, Farooqui KJ, Siddiqui MA, Khan AR. Prevalence of autoantibodies and risk estimation of development of youth onset type 1 diabetes in northern India. Diabetes Metab Syndr Clin Res Rev [Internet]. 2008 Feb;2(1):59–64. Available from: https://linkinghub.elsevier.com/retrieve/pii/S1871402107001014

82. Basu M, Pandit K, Banerjee M, Mondal S, Mukhopadhyay P, Ghosh S. Profile of auto-antibodies (Disease related and other) in children with type 1 diabetes. Indian J Endocrinol Metab [Internet]. 2020;24(3):256. Available from: http://www.ijem.in/text.asp?2020/24/3/256/288554

83. Al-Hassani N, Chedid F, Hadi S, Kaplan W. Prevalence of autoantibodies in type 1 diabetes patients and its association with the clinical presentation – UAE Eastern Region experience. J Pediatr Endocrinol Metab [Internet]. 2014 Jan 9; Available from: https://www.degruyter.com/document/doi/10.1515/jpem-2013-0430/html

84. Alyafei F, Soliman A, Alkhalaf F, Sabt A, De Sanctis V, Elsayed N, et al. Prevalence of β-cell antibodies and associated autoimmune diseases in children and adolescents with type 1 diabetes (T1DM) versus type 2 diabetes (T2DM) in Qatar. Acta Biomed [Internet]. 2018;89(S5):32–9. Available from: http://www.ncbi.nlm.nih.gov/pubmed/30049930

85. Andersson C, Vaziri-Sani F, Delli A, Lindblad B, Carlsson A, Forsander G, et al. Triple specificity of ZnT8 autoantibodies in relation to HLA and other islet autoantibodies in childhood and adolescent type 1 diabetes. Pediatr Diabetes [Internet]. 2013 Mar;14(2):97–105. Available from: https://onlinelibrary.wiley.com/doi/10.1111/j.1399-5448.2012.00916.x

86. Andersson C, Kolmodin M, Ivarsson S-A, Carlsson A, Forsander G, Lindblad B, et al. Islet cell antibodies (ICA) identify autoimmunity in children with new onset diabetes mellitus negative for other islet cell antibodies. Pediatr Diabetes [Internet]. 2014 Aug;15(5):336–44. Available from: https://onlinelibrary.wiley.com/doi/10.1111/pedi.12093

87. Awa WL, Boehm BO, Kapellen T, Rami B, Rupprath P, Marg W, et al. HLA-DR genotypes influence age at disease onset in children and juveniles with type 1 diabetes mellitus. Eur J Endocrinol [Internet]. 2010 Jul;163(1):97–104. Available from: https://eje.bioscientifica.com/view/journals/eje/163/1/97.xml

88. Bahendeka S, Wesonga R, Were TP, Nyangabyaki C. Autoantibodies and HLA class II DR-DQ genotypes in Ugandan children and adolescents with type 1 diabetes mellitus. Int J Diabetes Dev Ctries [Internet]. 2019 Jan 26;39(1):39–46. Available from: http://link.springer.com/10.1007/s13410-018-0622-5

89. Balasubramanian K, Dabadghao P, Bhatia V, Colman PG, Gellert SA, Bharadwaj U, et al. High Frequency of Type 1B (Idiopathic) Diabetes in North Indian Children With Recent-Onset Diabetes. Diabetes Care [Internet]. 2003 Sep 1;26(9):2697–2697. Available from: http://care.diabetesjournals.org/cgi/doi/10.2337/diacare.26.9.2697

90. Balcha SA, Demisse AG, Mishra R, Vartak T, Cousminer DL, Hodge KM, et al. Type 1 diabetes in Africa: an immunogenetic study in the Amhara of North-West Ethiopia. Diabetologia [Internet]. 2020 Oct 23;63(10):2158–68. Available from: http://www.ncbi.nlm.nih.gov/pubmed/32705316

91. Batstra MR, Pina M, Quan J, Mulder P, de Beaufort CE, Bruining GJ, et al. Fluctuations in GAD65 Antibodies After Clinical Diagnosis of IDDM in Young Children. Diabetes Care [Internet]. 1997 Apr 1;20(4):642–4. Available from: http://care.diabetesjournals.org/cgi/doi/10.2337/diacare.20.4.642

92. BETTERLE C, FUSARI A, PRESOTTO F, DAL PRA C, PEDINI B, LAZZAROTTO F, et al. Pancreatic Autoantibodies in Italian Patients with Newly Diagnosed Type 1 Diabetes Mellitus under the Age of 20 Years. Ann N Y Acad Sci [Internet]. 2006 Jan 24;958(1):271–5. Available from: https://onlinelibrary.wiley.com/doi/10.1111/j.1749-6632.2002.tb02985.x

93. Delli AJ, Vaziri-Sani F, Lindblad B, Elding-Larsson H, Carlsson A, Forsander G, et al. Zinc Transporter 8 Autoantibodies and Their Association With SLC30A8 and HLA-DQ Genes Differ Between Immigrant and Swedish Patients With Newly Diagnosed Type 1 Diabetes in the Better Diabetes Diagnosis Study. Diabetes [Internet]. 2012 Oct 1;61(10):2556–64. Available from: http://diabetes.diabetesjournals.org/cgi/doi/10.2337/db11-1659

94. Dotta F, Falorni A, Tiberti C, Dionisi S, Anastasi E, Torresi P, et al. Autoantibodies to the GM2-1 Islet Ganglioside and to GAD-65 at Type 1 Diabetes Onset. J Autoimmun [Internet]. 1997 Dec;10(6):585–8. Available from: https://linkinghub.elsevier.com/retrieve/pii/S0896841197901669

95. Bilbao JR, Rica I, Vázquez JA, Busturia MA, Castaño L. Influence of sex and age at onset on autoantibodies against insulin, GAD65 and IA2 in recent onset type 1 diabetic patients. Horm Res [Internet]. 2000;54(4):181–5. Available from: http://www.ncbi.nlm.nih.gov/pubmed/11416235

96. Al Alwan I, Bin Dajim N, Jawdat D, Tamim W, Al Ahmdi R, Albuhairan F. Prevalence of autoantibodies in children newly diagnosed with type 1 diabetes mellitus. Br J Biomed Sci [Internet]. 2012 Jan 20;69(1):31–3. Available from: https://www.tandfonline.com/doi/full/10.1080/09674845.2012.11669919

97. Carla B, Danila B, Paolo C, Patrizia PI, Riccardo S, Antonella M, et al. Clinical Presentation and Autoimmune Characteristics of Very Young Children at the Onset of Type 1 Diabetes Mellitus. J Pediatr Endocrinol Metab [Internet]. 2010 Jan;23(11). Available from: https://www.degruyter.com/document/doi/10.1515/jpem.2010.180/html

98. Borg H, Marcus C, Sjöblad S, Fernlund P, Sundkvist G. Insulin autoantibodies are of less value compared with islet antibodies in the clinical diagnosis of autoimmune type 1 diabetes in children older than 3 yr of age. Pediatr Diabetes [Internet]. 2002 Sep;3(3):149–54. Available from: http://doi.wiley.com/10.1034/j.1399-5448.2002.30305.x

99. Cedillo M, Libman IM, Arena VC, Zhou L, Trucco M, Ize-Ludlow D, et al. Obesity, Islet Cell Autoimmunity, and Cardiovascular Risk Factors in Youth at Onset of Type 1 Autoimmune Diabetes. J Clin Endocrinol Metab [Internet]. 2015 Jan;100(1):E82–6. Available from: https://academic.oup.com/jcem/article-lookup/doi/10.1210/jc.2014-2340

100. Chang Y-H, Shiau M-Y, Tsai S-T, Lan MS. Autoantibodies against IA-2, GAD, and topoisomerase II in type 1 diabetic patients. Biochem Biophys Res Commun [Internet]. 2004 Jul;320(3):802–9. Available from: https://linkinghub.elsevier.com/retrieve/pii/S0006291X0401263X

101. Chen Q-Y, Rowley MJ, Byrne GC, Jones TW, Tuomi T, Knowles WJ, et al. Antibodies to Glutamic Acid Decarboxylase in Australian Children with Insulin-Dependent Diabetes Mellitus and Their First-Degree Relatives. Pediatr Res [Internet]. 1993 Dec;34(6):785–90. Available from: http://www.nature.com/doifinder/10.1203/00006450-199312000-00018

102. Delli AJ, Lindblad B, Carlsson A, Forsander G, Ivarsson S-A, Ludvigsson J, et al. Type 1 diabetes patients born to immigrants to Sweden increase their native diabetes risk and differ from Swedish patients in HLA types and islet autoantibodies. Pediatr Diabetes [Internet]. 2010 Dec;11(8):513–20. Available from: https://onlinelibrary.wiley.com/doi/10.1111/j.1399-5448.2010.00637.x

103. El‐Amir MI, El‐Feky MA, Laine A, Härkönen T, El‐Badawy O, Eltayeb AA, et al. Risk genes and autoantibodies in Egyptian children with type 1 diabetes – low frequency of autoantibodies in carriers of the HLA‐DRB1*04:05‐DQA1*03‐DQB1*02 risk haplotype. Diabetes Metab Res Rev [Internet]. 2015 Mar 24;31(3):287–94. Available from: https://onlinelibrary.wiley.com/doi/10.1002/dmrr.2609

104. Elamin A, Mohamed Ibrahim Ali O, Tuvemo T. Islet-cell antibodies and endogenous insulin secretion in Sudanese diabetic children. Diabetes Res Clin Pract [Internet]. 1992 May;16(2):91–6. Available from: http://www.ncbi.nlm.nih.gov/pubmed/1600856

105. Garnier L, Marchand L, Benoit M, Nicolino M, Bendelac N, Wright C, et al. Screening of ZnT8 autoantibodies in the diagnosis of autoimmune diabetes in a large French cohort. Clin Chim Acta [Internet]. 2018 Mar;478:162–5. Available from: http://www.ncbi.nlm.nih.gov/pubmed/29288641

106. Elfving M, Lindberg B, Lynch K, Månsson M, Sundkvist G, Lernmark Å, et al. Number of islet autoantibodies present in newly diagnosed type 1 diabetes children born to non-diabetic mothers is affected by islet autoantibodies present at birth. Pediatr Diabetes [Internet]. 2008 Apr;9(2):127–34. Available from: https://onlinelibrary.wiley.com/doi/10.1111/j.1399-5448.2007.00349.x

107. Evia-Viscarra ML, Guardado-Mendoza R, Rodea-Montero ER. Clinical and Metabolic Characteristics among Mexican Children with Different Types of Diabetes Mellitus. Pietropaolo M, editor. PLoS One [Internet]. 2016 Dec 16;11(12):e0168377. Available from: https://dx.plos.org/10.1371/journal.pone.0168377

108. Fabris M, Zago S, Liguori M, Trevisan MT, Zanatta M, Comici A, et al. Anti-zinc transporter protein 8 autoantibodies significantly improve the diagnostic approach to type 1 diabetes: an Italian multicentre study on paediatric patients. Autoimmun Highlights [Internet]. 2015 Aug 21;6(1–2):17–22. Available from: https://autoimmunhighlights.biomedcentral.com/articles/10.1007/s13317-015-0068-4

109. Fajardo C, Piñón F, Carmona E, Sánchez-Cuenca JM, Merino JF, Carlés C. Influence of age on clinical and immunological characteristics of newly diagnosed type 1 diabetic patients. Acta Diabetol [Internet]. 2001 Mar 1;38(1):31–6. Available from: http://link.springer.com/10.1007/s005920170026

110. Fakhfakh R, Haddouk S, Hadj Hamida YB, Kamoun T, Ayed MB, Hachicha M, et al. Pancreatic autoantibodies in Tunisian children with newly diagnosed type 1 diabetes. Pathol Biol (Paris) [Internet]. 2008 May;56(3):130–2. Available from: http://www.ncbi.nlm.nih.gov/pubmed/18178035

111. Feeney SJ, Myers MA, Mackay IR, Zimmet PZ, Howard N, Verge CF, et al. Evaluation of ICA512As in Combination With Other Islet Cell Autoantibodies at the Onset of IDDM. Diabetes Care [Internet]. 1997 Sep 1;20(9):1403–7. Available from: http://www.ncbi.nlm.nih.gov/pubmed/9283787

112. Fida S, Myers M, Mackay I., Zimmet P., Mohan V, Deepa R, et al. Antibodies to diabetes-associated autoantigens in Indian patients with Type 1 diabetes: prevalence of anti-ICA512/IA2 and anti-SOX13. Diabetes Res Clin Pract [Internet]. 2001 Jun;52(3):205–11. Available from: https://linkinghub.elsevier.com/retrieve/pii/S0168822701002303

113. Forbes L V., Scott RS, Brown LJ, Darlow BA. Immunogenetic, Clinical, and Demographic Characterization of Childhood Type I Diabetes in New Zealand. Diabetes Care [Internet]. 1995 Nov 1;18(11):1428–33. Available from: http://care.diabetesjournals.org/cgi/doi/10.2337/diacare.18.11.1428

114. Adojaan B, Sabbah E, Vähäsalo P, Åkerblom HK, Podar Τ, Knip M. Glutamic Acid Decarboxylase and Islet Cell Antibodies in Healthy Estonian Children. J Pediatr Endocrinol Metab [Internet]. 1999 Jan;12(5). Available from: https://www.degruyter.com/document/doi/10.1515/JPEM.1999.12.5.667/html

115. Bonifacio E, Genovese S, Braghi S, Bazzigaluppi E, Lampasona V, Bingley PJ, et al. Islet autoantibody markers in IDDM: risk assessment strategies yielding high sensitivity. Diabetologia [Internet]. 1995 Jul;38(7):816–22. Available from: http://www.ncbi.nlm.nih.gov/pubmed/7556984

116. Ilonen J, Lempainen J, Hammais A, Laine A-P, Härkönen T, Toppari J, et al. Primary islet autoantibody at initial seroconversion and autoantibodies at diagnosis of type 1 diabetes as markers of disease heterogeneity. Pediatr Diabetes [Internet]. 2018;19(2):284–92. Available from: http://www.ncbi.nlm.nih.gov/pubmed/28597949

117. Jacobsen LM, Larsson HE, Tamura RN, Vehik K, Clasen J, Sosenko J, et al. Predicting progression to type 1 diabetes from ages 3 to 6 in islet autoantibody positive TEDDY children. Pediatr Diabetes [Internet]. 2019;20(3):263–70. Available from: http://www.ncbi.nlm.nih.gov/pubmed/30628751

118. Pöllänen PM, Ryhänen SJ, Toppari J, Ilonen J, Vähäsalo P, Veijola R, et al. Dynamics of Islet Autoantibodies During Prospective Follow-Up From Birth to Age 15 Years. J Clin Endocrinol Metab [Internet]. 2020;105(12). Available from: http://www.ncbi.nlm.nih.gov/pubmed/32882033

119. Vehik K, Haller MJ, Beam CA, Schatz DA, Wherrett DK, Sosenko JM, et al. Islet Autoantibody Seroconversion in the DPT-1 Study: mtification for repeat screening throughout childhood. Diabetes Care [Internet]. 2011 Feb 1;34(2):358–62. Available from: http://care.diabetesjournals.org/cgi/doi/10.2337/dc10-1494

120. Mobasseri M, Shirmohammadi M, Amiri T, Vahed N, Hosseini Fard H, Ghojazadeh M. Prevalence and incidence of type 1 diabetes in the world: a systematic review and meta-analysis. Heal Promot Perspect [Internet]. 2020 Mar 30;10(2):98–115. Available from: http://hpp.tbzmed.ac.ir/Article/hpp-31920

121. Pociot F, Nørgaard K, Hobolth N, Andersen O, Nerup J. A nationwide population-based study of the familial aggregation of type 1 (insulin-dependent) diabetes mellitus in Denmark. Danish Study Group of Diabetes in Childhood. Diabetologia [Internet]. 1993 Sep;36(9):870–5. Available from: http://www.ncbi.nlm.nih.gov/pubmed/8405760

122. Crotti C, Selmi C. Glutamic Acid Decarboxylase Antibody. In: Autoantibodies [Internet]. Third Edit. Elsevier; 2014. p. 385–9. Available from: https://linkinghub.elsevier.com/retrieve/pii/B9780444563781000460

123. Pihoker C, Gilliam LK, Hampe CS, Lernmark A. Autoantibodies in diabetes. Diabetes [Internet]. 2005 Dec;54 Suppl 2(December):S52-61. Available from: http://www.ncbi.nlm.nih.gov/pubmed/16306341

124. Ilonen J, Lempainen J, Hammais A, Laine A-P, Härkönen T, Toppari J, et al. Primary islet autoantibody at initial seroconversion and autoantibodies at diagnosis of type 1 diabetes as markers of disease heterogeneity. Pediatr Diabetes [Internet]. 2018 Mar;19(2):284–92. Available from: https://onlinelibrary.wiley.com/doi/10.1111/pedi.12545

125. Kulmala P, Savola K, Petersen JS, Vähäsalo P, Karjalainen J, Löppönen T, et al. Prediction of insulin-dependent diabetes mellitus in siblings of children with diabetes: A population-based study. J Clin Invest. 1998;101(2):327–36.
